# A peculiar new species of the genus *Tetrasticta* Kraatz (Coleoptera, Staphylinidae, Aleocharinae) from Peninsular Malaysia

**DOI:** 10.3897/zookeys.336.5382

**Published:** 2013-09-27

**Authors:** Shûhei Yamamoto, Munetoshi Maruyama

**Affiliations:** 1 Entomological Laboratory, Graduate School of Bioresource and Bioenvironmental Sciences, Kyushu University, Hakozaki 6-10-1, Fukuoka, 812-8581 Japan; 2 The Kyushu University Museum, Hakozaki 6-10-1, Fukuoka, 812-8581 Japan

**Keywords:** Aleocharini, description, Oriental region, taxonomy, under bark

## Abstract

*Tetrasticta gnatha* **sp. n.**, collected under the bark of a rotten fallen tree in Peninsular Malaysia, is described. A habitus photograph, line drawings of diagnostic characters, and a diagnosis are provided. The new species is readily distinguished from all known congeners by having long mandibles, and long, curved maxillary palpi.

## Introduction

The staphylinid genus *Tetrasticta* Kraatz, 1857 (Aleocharinae: Aleocharini) is a small genus that currently contains ten species from the Oriental and Afrotropical regions (Maruyama and Sugaya 2002; Maruyama 2004; Pace 2008). *Tetrasticta* is distinguished from the other genera of the tribe Aleocharini Fleming, 1821 by having large eyes, thick antennae, a broad pronotum, short elytra, and a long, broad intercoxal-process of the metaventrite (see, Maruyama 2004). The life histories of most species are unknown, but some species, e.g., *Tetrasticta polita* Kraatz, 1857 and *Tetrasticta laeta* Maruyama & Sugaya, 2002, are presumed to be weakly integrated termitophiles (Kraatz 1857; Cameron 1939; Maruyama et al. 2013). A morphology-based phylogeny by Kanao et al. (2011) suggested that the termitophilous subtribe Compactopediina Kistner, 1970 of the Aleocharini evolved termitophily within the *Tetrasticta* genus group (sensu Maruyama 2004).

Previously, three species of *Tetrasticta* were known from Malaysia (Cameron 1943; Maruyama 2004; Pace 2008), one of which was recorded from Peninsular Malaysia (Maruyama 2004).

## Materials and methods

The technical procedures, terminology, and other methods used here are given in detail in Maruyama (2006) and Yamamoto and Maruyama (2012). In the descriptions, the numbers of macrosetae are those on one side of the body. The macrosetae on tergite VIII and sternite VIII are illustrated only on one side of the segments. All measurements in the paper are given in millimetres as follows: minimum length–maximum length (mean±SD). Most of the type specimens including the holotype are preserved at the Kyushu University Museum, Fukuoka, Japan.

## Taxonomy

### Genus *Tetrasticta* Kraatz

*Tetrasticta* Kraatz, 1857: 54 [original description]. See Maruyama (2004) for synonymic list.

#### Tetrasticta gnatha sp. n.

http://zoobank.org/9480CC00-367F-4F91-B88D-CE49D7F1FD4F

http://species-id.net/wiki/Tetrasticta_gnatha

Figs 1 – 12

##### Type material.

Holotype: male, “Nr. Kenyir Lake / Kuala Terengganu St. / W. MALAYSIA / 1-III-2002 / Tomoyuki TSURU leg.”. Paratypes: 1 male, 1 female, 9 unsexed specimens, same data as the holotype.

##### Description.

**Figure 1.**
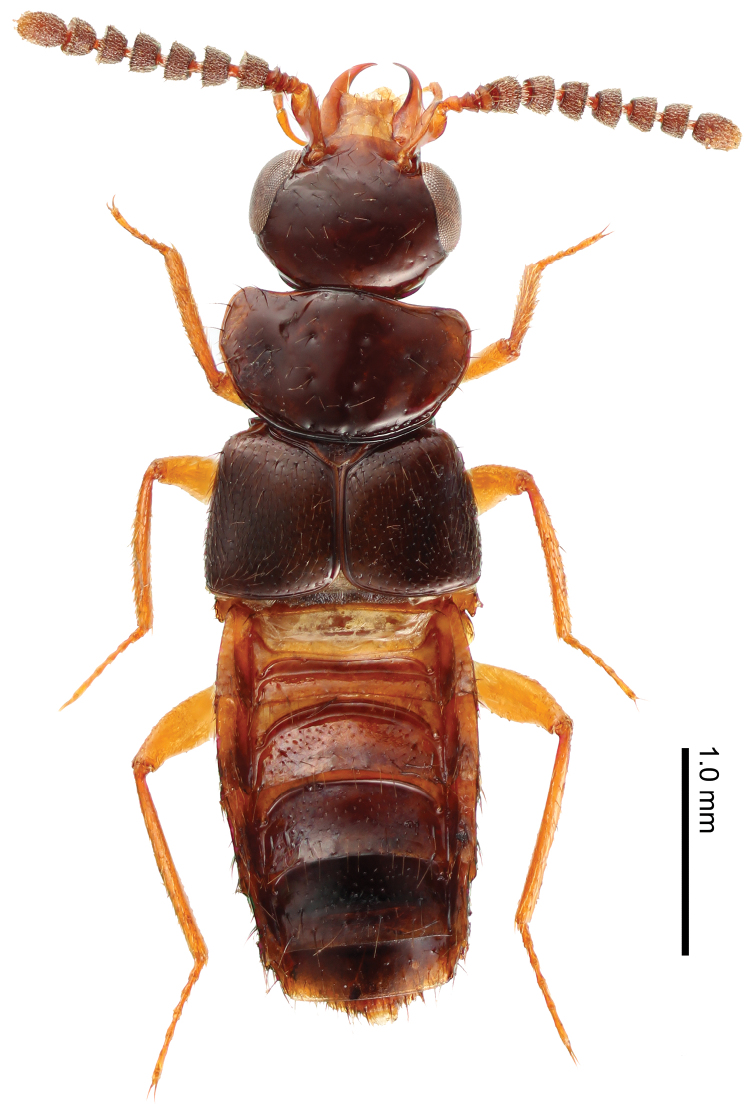
Habitus of *Tetrasticta gnatha* sp. n., female, dorsal view.

**Figures 2–4.**
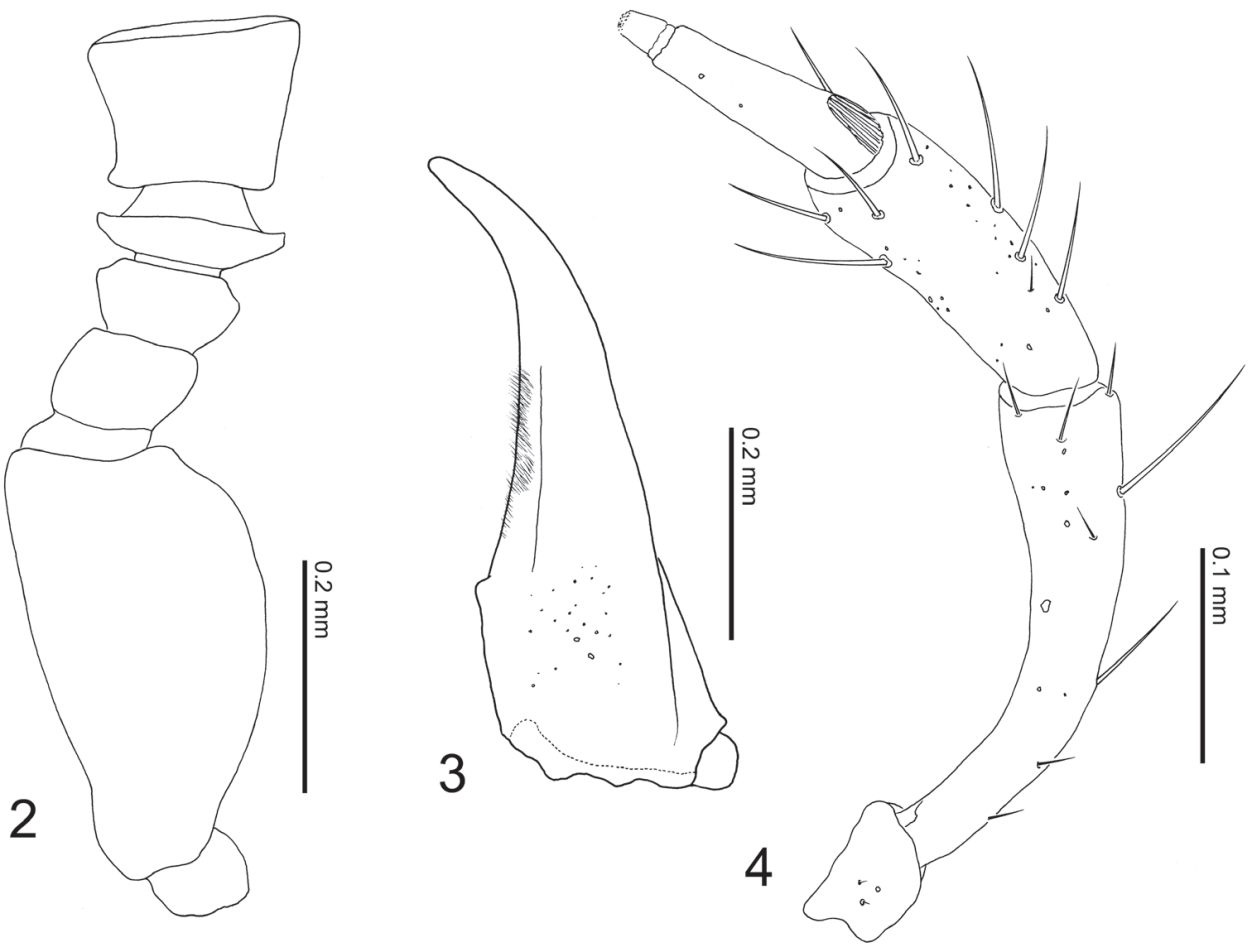
*Tetrasticta gnatha* sp. n. **2** antennal segments I–V (right, dorsolateral view) **3** right mandible, male, dorsal view (hairs along inner side are those of prostheca) **4** right maxillary palpus, male, ventral view.

**Body:** broad, somewhat flattened, shining (Fig. 1). **Color:** reddish brown; abdomen paler, with darker tergites V–VII; antennal segments I–IV, mouthparts, and legs reddish yellow; antennal segments V–XI brown. **Head:** large, as broad as pronotum, somewhat pentangular, moderately convex above, slightly wider than long; apical margin of clypeus rounded; frons produced, V-shaped; surface sparsely covered with minute, yellow setae; eyes prominent, length 0.64 times that of head. Antennae slightly longer than combined length of head and pronotum; segment I long, as long as combined length of segments II–V; segment II small, less than one-third of I; segment III small, shorter than II; segment IV extremely short, much wider than long, about half as long as III; segment V slightly wider than long; segments VI–X wider than long; segment XI oval, longer than wide; relative length of segments from base to apex: 31:7:6:3.5:9.5:10:10:10:10:10:22 (Fig. 2). **Mouthparts:** labrum much wider than long (W/L = 1.9), anterior margin widely emarginate. Mandibles slightly asymmetric, strongly curved, pointed apically (Fig. 3). Maxilla with a long and strongly curved maxillary palpus (Fig. 4). Mentum somewhat semicircular, much wider than long (W/L = 2.3); surface with 4 setae, and dozens of pores antero-medially. **Pronotum:** much wider than long, semicircular; disc with three pairs of small depressions (two pairs medially, one pair laterally); surface sparsely covered with yellow setae, and with approximately five bristles along lateral margin; each depression bearing a small bristle. **Elytra:** wider than long, subparallel-sided, rounded posterolaterally; surface moderately punctured and covered with yellow setae, and with four bristles laterally. **Legs:** rather short in length; relative lengths of tarsomeres from base to apex: 7:5:5:6:10.5 in foretarsus; 10:6:7:7:11 in midtarsus; 11:11:11:11:19 in hindtarsus. **Abdomen:** flattened, subpararell-sided, widest around segments IV and V; tergites III–VII sparsely covered with small setae.

**Figures 5–12.**
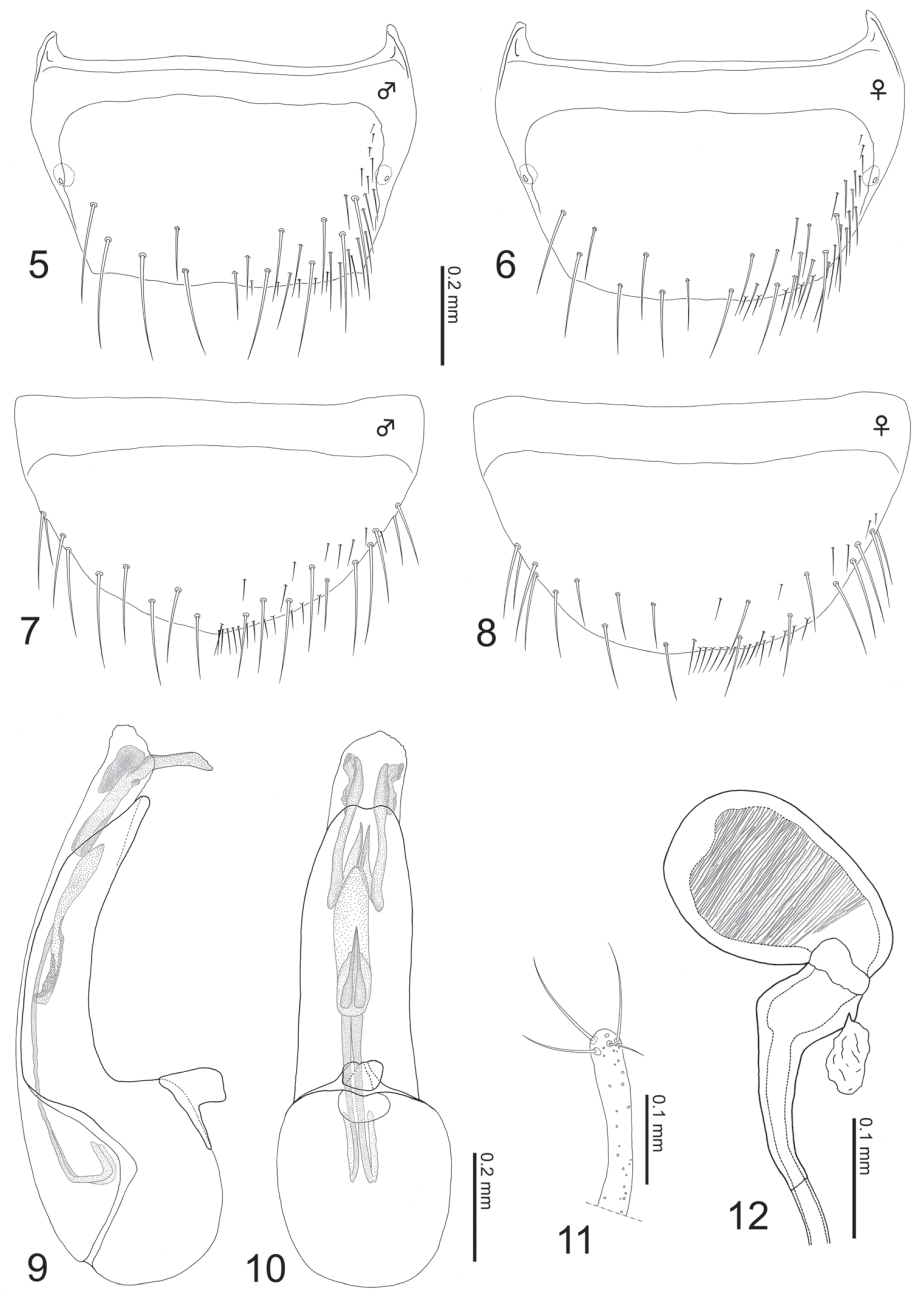
*Tetrasticta gnatha* sp. n. **5** male tergite VIII, dorsal view **6** female tergite VIII, dorsal view **7** male sternite VIII, ventral view **8** female sternite VIII, ventral view **9** median lobe of aedeagus, lateral view **10** ditto, ventral view **11** apical lobe of paramerite **12** spermatheca.

**Male:** tergite VIII generalized in shape; surface smooth, with approximately 5–6 macrosetae (Fig. 5). Sternite VIII generalized in shape, semicircular in dorsal view, with approximately 9–10 macrosetae (Fig. 7). Median lobe of aedeagus slightly narrowed apically in lateral view; inner sac with flagellum of copulatory piece not coiled (Figs 9–10); apical lobe of paramerite long, slightly dilated apically with four setae (Fig. 11).

**Female:** tergite VIII generalized as in male, with approximately 7–9 macrosetae (Fig. 6). Sternite VIII generalized as in male, with approximately 9–10 macrosetae (Fig. 7). Spermatheca: moderately curved; border between basal and apical portions narrowly membranous; basal portion dilated apically, longer than apical portion, with prominent opening of spermathecal gland; apical portion oval, inner wall of apical part three-quarters to four-fifths densely striate (Fig. 12).

**Measurements.** BL, ca. 3.7–5.6 (4.4±0.5); HL, 0.70–0.91 (0.79±0.06); HW, 0.99–1.15 (1.07±0.06); AL, 1.68–1.97 (1.84±0.09); PL, 0.71–0.84 (0.77±0.05); PW, 1.12–1.36 (1.25±0.07); EL, 0.61–0.78 (0.70±0.05); EW, 1.22–1.56 (1.38±0.09); FTL, 0.64–0.81 (0.72±0.05); MTL, 0.78–0.97 (0.89±0.06); HTL, 0.89–1.16 (1.04±0.07); HW/HL, 1.22–1.52 (1.34±0.09); PW/PL, 1.58–1.68 (1.63±0.03). *N* = 12.

##### Diagnosis.

This new species is easily distinguished from other *Tetrasticta* species by having a large head, which is as wide as pronotum, flattened body with unique mouthparts (i.e., strongly curved maxillary palpi, especially segment II, and curved, sharply pointed mandibles). Furthermore, the species lacks a coiled flagellum inside the median lobe of the male aedeagus.

##### Bionomics.

Dr. T. Tsuru collected all the specimens from a rotten log about 50 cm in diameter lying in the rainforest.

##### Distribution.

Malaysia (Peninsula).

##### Remarks.

*Tetrasticta* belongs to the *Tetrasticta* genus group, which comprises the genera *Creochara* Cameron, 1931, *Cratoacrochara* Pace, 1986, *Ilarochara* Pace, 1993, *Aleonictus* Kistner, 1997, *Formicaenictus* Kistner, 1997, and *Myrmecosticta* Maruyama, 2011 (Maruyama 2004; Maruyama et al. 2011). Pace (2010) synonymized *Creochara* with *Tetrasticta*, but did not provide an appropriate explanation; we do not follow this concept here. *Tetrasticta gnatha* is well characterized by the following character states: 1) long mandibles; 2) long, 3) curved segment II of the maxillary palpus; and 4) short, 5) simple flagellum of the median lobe of the aedeagus. Of these, states 2) and 3) are probably correlated with long mandibles, as also observed in the other aleocharines with long mandibles (e.g., some Lomechusini species). All other known species of *Tetrasticta* share a long, coiled flagellum of the median lobe of the aedeagus. The other character states fully coincide with those of *Tetrasticta*. A short, simple flagellum is also observed in *Aleonictus* and *Formicaenictus* in the same genus group, which are closely allied to *Tetrasticta*, but this state is apparently plesiomorphic, and cannot support a relationship between *Tetrasticta gnatha* and these genera. Although *Tetrasticta gnatha* is unique within the *Tetrasticta* genus group at first glance, we do not erect a genus for it. All of the type specimens of *Tetrasticta gnatha* were found under bark. Since no behavioral observations were made, termitophily of *Tetrasticta gnatha* remain uncertain. The long mandibles in both sexes and the flattened body suggest a predatory life under bark.

##### Etymology.

The Greek *gnathos* means jaw, for the exceptionally long mandibles.

## Supplementary Material

XML Treatment for Tetrasticta gnatha
